# Pretreatment clinical and hematologic prognostic factors of metastatic urothelial carcinoma treated with pembrolizumab: a systematic review and meta-analysis

**DOI:** 10.1007/s10147-021-02061-0

**Published:** 2021-11-10

**Authors:** Takafumi Yanagisawa, Keiichiro Mori, Satoshi Katayama, Hadi Mostafaei, Fahad Quhal, Ekaterina Laukhtina, Pawel Rajwa, Reza Sari Motlagh, Abdulmajeed Aydh, Frederik König, Nico C. Grossmann, Benjamin Pradere, Jun Miki, Takahiro Kimura, Shin Egawa, Shahrokh F. Shariat

**Affiliations:** 1 Department of Urology, Comprehensive Cancer Center, Medical University of Vienna, Wahringer Gurtel 43 18-20, 1090 Vienna, Austria; 2 Department of Urology, The Jikei University School of Medicine, Tokyo, Japan; 3 Department of Urology, Okayama University Graduate School of Medicine, Dentistry and Pharmaceutical Sciences, Okayama, Japan; 4 Research Center for Evidence Based Medicine, Tabriz University of Medical Sciences, Tabriz, Iran; 5 Department of Urology, King Fahad Specialist Hospital, Dammam, Saudi Arabia; 6 Institute for Urology and Reproductive Health, Sechenov University, Moscow, Russia; 7 Department of Urology, Medical University of Silesia, Zabrze, Poland; 8 Men’s Health and Reproductive Health Research Center, Shahid Beheshti University of Medical Sciences, Tehran, Iran; 9 Department of Urology, King Faisal Medical City, Abha, Saudi Arabia; 10 Department of Urology, University Medical Centre Hamburg-Eppendorf, Hamburg, Germany; 11 Department of Urology, University Hospital Zurich, Zurich, Switzerland; 12 Department of Urology, Luzerner Kantonsspital, Lucerne, Switzerland; 13 Division of Urology, Department of Special Surgery, Hourani Center for Applied Scientific Research, Al-Ahliyya Amman University, Amman, Jordan; 14 Department of Urology, University of Texas Southwestern Medical Center, Dallas, TX USA; 15 Department of Urology, Second Faculty of Medicine, Charles University, Prague, Czech Republic; 16 Department of Urology, Weill Cornell Medical College, New York, NY USA; 17 Karl Landsteiner Institute of Urology and Andrology, Vienna, Austria

**Keywords:** Metastatic urothelial carcinoma, Pembrolizumab, Prognostic factor

## Abstract

Pembrolizumab is the standard for the first and second lines in treating metastatic urothelial carcinoma (UC). This systematic review and meta-analysis aimed to assess the value of pretreatment clinical characteristics and hematologic biomarkers for prognosticating response to pembrolizumab in patients with metastatic UC. PUBMED^®^, Web of Science™, and Scopus^®^ databases were searched for articles published before May 2021 according to the PRISMA (Preferred Reporting Items for Systematic Review and Meta-Analyses) statement. Studies were deemed eligible if they evaluated overall survival (OS) in patients with metastatic urothelial carcinoma treated with pembrolizumab and pretreatment clinical characteristics or laboratory examination. Overall, 13 studies comprising 1311 patients were eligible for the meta-analysis. Several pretreatment patients’ demographics and hematologic biomarkers were significantly associated with worse OS as follows: Eastern Cooperative Oncology Group Performance Status (ECOG-PS) ≥ 2 (Pooled hazard ratio [HR]: 3.24, 95% confidence interval [CI] 2.57–4.09), presence of visceral metastasis (Pooled HR: 1.84, 95% CI 1.42–2.38), presence of liver metastasis (Pooled HR: 4.23, 95% CI 2.18–8.20), higher neutrophil–lymphocyte ratio (NLR) (Pooled HR: 1.29, 95% CI 1.07–1.55) and, higher c-reactive protein (CRP) (Pooled HR: 2.49, 95% CI 1.52–4.07). Metastatic UC patients with poor PS, liver metastasis, higher pretreatment NLR and/or CRP have a worse survival despite pembrolizumab treatment. These findings might help to guide the prognostic tools for clinical decision-making; however, they should be interpreted carefully, owing to limitations regarding the retrospective nature of primary data.

**Supplementary Information:**

The online version contains supplementary material available at 10.1007/s10147-021-02061-0.

## Introduction

Urothelial carcinomas (UCs) located in the lower (bladder and urethra) or the upper (renal pelvicalyceal system and ureter) urinary tract are the 6th most common tumors in developed countries [1]. In recent years, immune checkpoint inhibitors (ICIs) targeting programmed cell death protein 1 (PD-1) and programmed death-ligand 1 (PD-L1) have been used in patients with locally advanced or metastatic urothelial carcinomas (mUCs) [2]. Pembrolizumab and nivolumab as PD-1 inhibitors, atezolizumab, avelumab, and durvalumab as PD-L1 inhibitors have been approved by the U.S. Food and Drug Administration. However, only pembrolizumab demonstrated significant overall survival benefit in a phase III Randomized Control Trial (RCT) [3]. Therefore, in the EAU guidelines, pembrolizumab is recommended to offer patients in the second-line mUC setting (i.e., post-platinum) [4].

Despite the advances offered by ICIs, the objective response rate (ORR) of pembrolizumab is around 20% in first- and second-line mUC [3, 5]. The development of predictive biomarkers is indispensable for patient selection, specifically with the avenue of multiple novel therapeutic options such as combination therapies and targeted therapies [6–9]. For intra-tumoral biomarkers, expression of PD-1 ligand PD-L1 has been found to exhibit more or less some predictive value for anti-PD-1-directed therapy in various cancers [10–13]. However, the utility of PD-L1 expression status in patients with metastatic UCs remains controversial and unclear [14–21]. Other biomarkers helping to predict the likelihood of response to anti-PD-1-directed therapy, including immunohistochemical biomarkers, molecular subtyping, immune gene expression analysis by RNA sequencing, mutations in DNA damage repair genes, and tumor mutational burden, have been tested [14, 22–24]. However, these biomarkers remain suboptimal for clinical application due to technical issues and suffer from the complexity underlying each tumor and temporal as well as spatial heterogeneity.

Therefore, clinical prognostic factors, which are easy to use based on reliable, widely available parameters, are crucial for assessing the result of clinical trials and guiding clinical decision-making. In patients treated with first-line chemotherapy for metastatic UCs, poor performance status (PS), visceral metastases, number of visceral metastases, leukocyte count, and low hemoglobin have been demonstrated as independent prognostic factors [25–30]. For patients treated with salvage chemotherapy-refractory after platinum-based combination chemotherapy, prognostic factors were consistent with the previous report [31]. However, these prognostic factors have not been validated in the context of novel agents, including ICIs.

Therefore, this systematic review and meta-analysis were conducted to evaluate and assess the pretreatment prognostic factors and oncologic outcomes following pembrolizumab for metastatic UCs as 2nd line therapy after platinum-based combination chemotherapy.

## Methods

The protocol has been registered in the International Prospective Register of Systematic Reviews database (PROSPERO: CRD42021258811).

### Search strategy

This meta-analysis was carried out based on the guidelines of the Preferred Reporting Items for Meta-Analyses of Observational Studies in Epidemiology Statement (Supplementary Fig. 1) [32]. In May 2021, a literature search on PUBMED^®^, Web of Science™, and Scopus^®^ databases was performed to identify reports that investigated the prognostic value of clinical and hematologic factors in patients with metastatic UC treated with pembrolizumab. The keywords used in our search strategy were as follows: (bladder cancer) OR (bladder carcinoma) OR (urothelial cancer) OR (urothelial carcinoma) AND (advanced OR metastatic) AND (pembrolizumab). The primary outcome of interest was overall survival (OS). Initial screening was performed independently by two investigators based on the titles and abstracts to identify ineligible reports. Potentially relevant reports were subjected to a full-text review. Additionally, reference lists of the retrieved articles were analyzed to identify further studies. Disagreements were resolved by consensus with the additional investigators.

### Inclusion and exclusion criteria

Studies were included if they investigated 2nd line metastatic UC patients with pretreatment clinical or hematological abnormal factors (Patients) who were treated with pembrolizumab (Interventions) compared to those without pretreatment clinical or hematological abnormal factors (Comparisons) to assess the independent predictive value of clinical and hematological factors on OS (Outcome) utilizing multivariate Cox regression analysis (Study design) in non-randomized observational, randomized, or cohort studies.

Studies lacking original patient data, reviews, letters, editorial comments, meeting abstracts, replies from authors, case reports, and non-English articles were excluded. Studies in the neoadjuvant/adjuvant setting, 1st line metastatic UC setting, and combination with chemotherapy were also excluded. In cases of duplicate publications, the higher quality or the most recent publication was selected.

### Data extraction

Data were extracted independently by two authors. First author’s name, publication year, recruitment country and institution, patient recruitment period, number of patients, age, sex, study design, follow-up duration, primary site, metastatic site, objective response rate (ORR), clinical characteristics, and hematologic biomarker were retrieved. Subsequently, the hazard ratios (HR) and 95% confidence intervals (CI) of pretreatment prognostic factors associated with OS were retrieved. The HRs were extracted from the multivariate analyses.

### Risk of bias assessment

As all included studies were non-randomized observational studies, assessment of study quality and risk of bias was performed using the Risk Of Bias In Non-randomized Studies of Interventions (ROBINS-I) tool following the Cochrane Handbook for Systematic Reviews of Interventions. Each bias domain and overall risk of bias was judged as 'Low', 'Moderate', 'Serious' or 'Critical' risk of bias. The main confounders were identified as the critical prognostic factors of OS. The presence of confounders was determined by consensus and review of the literature. The ROBINS-I assessment of each study was performed independently by two authors. (Supplementary Table 1).

### Statistical analyses

Forest plots were used to assess the multivariate HRs, to summarize them and, to describe the relationships between pretreatment clinical characteristics or hematologic biomarkers and OS. Studies were not considered in the meta-analysis if they used univariate Cox proportional hazard regression or general logistic regression analyses. Heterogeneity among the outcomes of included studies in this meta-analysis was evaluated using Cochrane’s *Q* test and the *I*^2^ statistic. When significant heterogeneity (*P* value of < 0.05 in the Cochrane *Q* test and a ratio > 50% in *I*^2^ statistics) was observed, a random-effects model was applied [33, 34]. Fixed-effects models for the calculation of pooled HRs for non-heterogeneous results were applied []. Funnel plots was used for assessment of publication bias (Supplementary Fig. 2). All analyses were conducted using Review Manager 5.3 (The Cochrane Collaboration, Copenhagen, Denmark), and the statistical significance level was set at *P* < 0.05.

## Results

### Study selection and characteristics

**Fig. 1:**
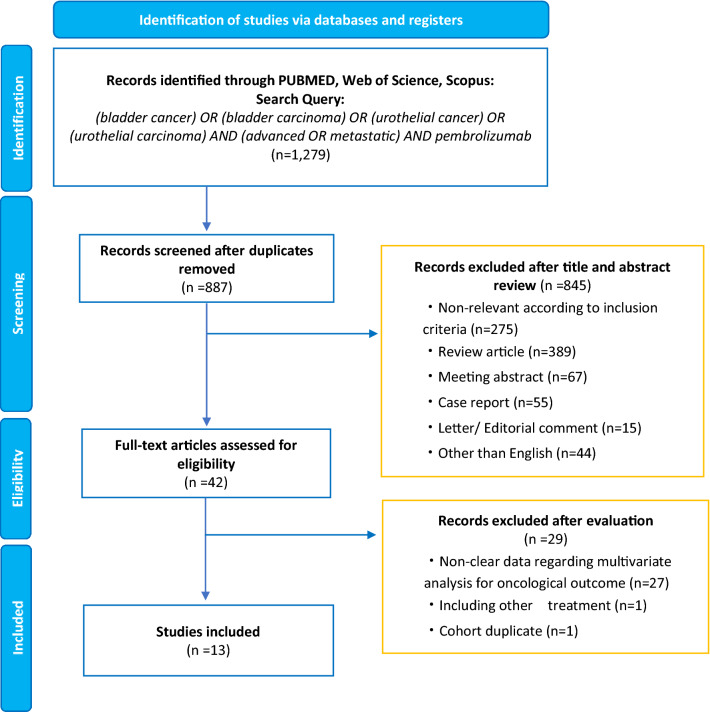
The preferred reporting items for systematic reviews and meta-analyses (PRISMA) flow chart, detailing the article selection process

Our initial search identified 1279 records. After removing duplicates, 887 records remained (Fig. 1). After screening the titles and abstracts, a full-text review was performed for 42 articles. Finally, we identified 13 studies comprising 1311 patients treated with pembrolizumab for cisplatin-refractory metastatic UCs according to our inclusion criteria [35–47]. The characteristics of included patients and the outcomes are shown in Table 1. All included studies were retrospective studies from Japan published between 2020 and 2021. The median age and follow-up range were from 70 to 74 years and 5.5 to 17.7 months, respectively. Of 1311 patients, 907 were male and 404 were female. The pooled rate of UTUC patients was 45.7% (range 35–69%), the pooled rate of liver metastasis was 21.2% (12–32%), and the pooled ORR was 25.6% (range 14.4–37%).

**Table 1:** Demographics of included studies

Study	Year	*N*	Nation	Period	Sex	Median age	Primary	Metastatic site	ORR	Follow-up (month)	Design	Significant clinical factor for OS (cut off)	Significant blood marker for OS (cut off)
Etani	2020	52	Japan	2018–2019	M:43 F:9	71	UTUC: 26 (50%) BC: 26 (50%)	LN: 21(40%) Lung: 16 (31%) Liver: 10 (19%)	21%	12.2	R	ECOG-PS (2) Visceral mets. GNRI (92)	None
Kijima	2020	97	Japan	2018–2019	M: 76 F: 21	70	UTUC: 40 (41%) BC: 57 (59%)	LN: 75 (77%) Lung: 38 (39%) Liver: 20 (21%) Bone: 13 (13%)	26.8%	5.5	R	ECOG-PS (2) Liver mets.	CRP (Non-responder)
Furubayashi	2020	34	Japan	2018–2019	M:28 F:6	71	UTUC: 12 (35%) BC: 13 (38%) Both: 9 (27%)	Visceral: 27 (79%) Liver: 11 (32%)	20.6%	7.7	R	Liver mets. Time from previous chemotherapy (≥ 3 mo.)	None
Kobayashi	2020	755 (Discovery cohort: *n* = 463)	Japan	2015–2019	M:357 F:106	71	UTUC: 179 (39%) BC: 230 (50%) Both: 27 (6%)	LN only: 156 (34%) Liver: 101 (22%) Other: 206 (44%)	※Low-risk: 48.3% Intermediate-risk 28.8% High-risk 10.5%	17.7	R	ECOG-PS (2) Visceral mets. Liver mets. Smoking history	Hb (11 g/dl) NLR (3)
Ogihara	2020	78	Japan	2017–2019	M: 44 F: 24	72.2 (mean)	UTUC: 35 (45%) BC: 43 (55%)	LN: 39 (50%) Lung: 28 (36%) Liver: 10 (13%) Bone: 16 (21%)	30%	7.4	R	N.A.	NLR (CSS) (3.5)
Shimizu	2020	27	Japan	2017–2019	M: 23 F: 4	73	UTUC: 12 (44%) BC: 15 (56%)	LN: 23 (85%) Lung: 15 (56%) Liver: 6 (22%) Bone: 2 (7%)	37%	7	R	Sarcopenia	NLR (PFS) (4)
Tamura	2020	41	Japan	2018–2019	M: 29 F: 12	70	UTUC: 22 (54%) BC: 19 (46%)	LN: 26 (63%) Lung: 15 (37%) Liver: 8 (20%) Bone: 7 (17%)	14.6%	6.2	R	ECOG-PS (2) Number of metastatic organs (2)	NLR change (3.68 + 6.12%),
Inoue	2020	73	Japan	2017–2019	M: 56 F: 17	72	UTUC: 41 (56%) BC: 27 (37%) Both: 5 (7%)	Lung: 34 (47%) Liver: 22 (30%) Bone: 11 (15%)	17.8%	5.5	R	irAE	N.A.
Kadono	2021	91	Japan	2018–2019	M: 65 F: 26	N.A.	N.A.	N.A.	N.A.	7.9	R	N.A.	NLR (2.9)
Fukuokaya	2021	95	Japan	2018–2020	M: 65 F: 30	72	UTUC: 51 (54%) BC: 44 (46%)	LN only: 40 (42%) Visceral: 55 (58%)	34.7% (iCR + iPR)	8.2	R	Smoking exposure (≥ 25 pack-years)	N.A.
Yamamoto	2021	121	Japan	2015–2019	M: 87 F: 34	74	UTUC: 56 (46%) BC: 51 (42%) Both: 14 (12%)	LN: 79 (65%) Lung: 49 (40%) Liver: 24 (20%) Bone: 25 (21%)	21.5%	7.9	R	ECOG-PS (2) Visceral mets.	CRP (0.56 mg/dl) NLR (3)
Fujiwara	2021	74	Japan	2018–2020	M:55 F:19	69	UTUC: 38 (51%) BC: 36 (49%)	LN: 57 (77%) Lung: 31 (42%) Liver: 9 (12%) Bone: 13 (18%)	30.2%	8.5	R	ECOG-PS (2) Liver mets.	LDH (ULN) CRP (0.5 mg/dl)
Ishiyama	2021	65	Japan	2018–2020	M: 44 F: 21	73	UTUC: 45 (69%) BC: 20 (31%)	LN: 45 (69%) Lung: 24 (37%) Liver: 18 (28%)	26.2%	7.2	R	ECOG-PS, UTUC, Low PNI	N.A.

*M* Male, *F* Female, *N* Number, *R* Retrospective, *ORR* Objective Response Rate, *OS* Overall Survival, *PFS* Progression-Free Survival, *CSS* Cancer-Specific Survival, *UTUC* Upper Urinary Tract Urothelial Carcinoma, *BC* Bladder Cancer, *LN* Lymph Node, *mets.* Metastasis, *GNRI* Geriatric Nutritional Risk Index, *ECOG-PS* Eastern Cooperative Oncology Group Performance Status, *Hb* Hemoglobin, *LDH* Lactate Dehydrogenase, *NLR* Neutrophil Lymphocyte Ratio, *N.A.* Not Applicable, *iCR* immune Complete Response, *iPR* immune Partial Response, *irAE* immune related Adverse Events, *ULN* Upper Limit of Normal, *PNI* Prognostic Nutritional Index, ※Risk classification defined as score sum of 4 variables: surgical removal of primary site no (1) or yes (0); smoking history yes(1) or no (0); NLR ≥ 3 (1) or < 3 (0); Hb < 11 g/dl (1) or ≥ 11 g/dl (0); metastasis in the liver (2), other organs (1), or lymph nodes only (0)and ECOG PS ≥ 2 (2), 1 (1), or 0 (0); Low-risk: 0–1, Intermediate-risk: 2–5, High-risk: 6

### Meta‑analysis

#### Association of ECOG-PS with OS in mUC treated with pembrolizumab

**Fig. 2:**
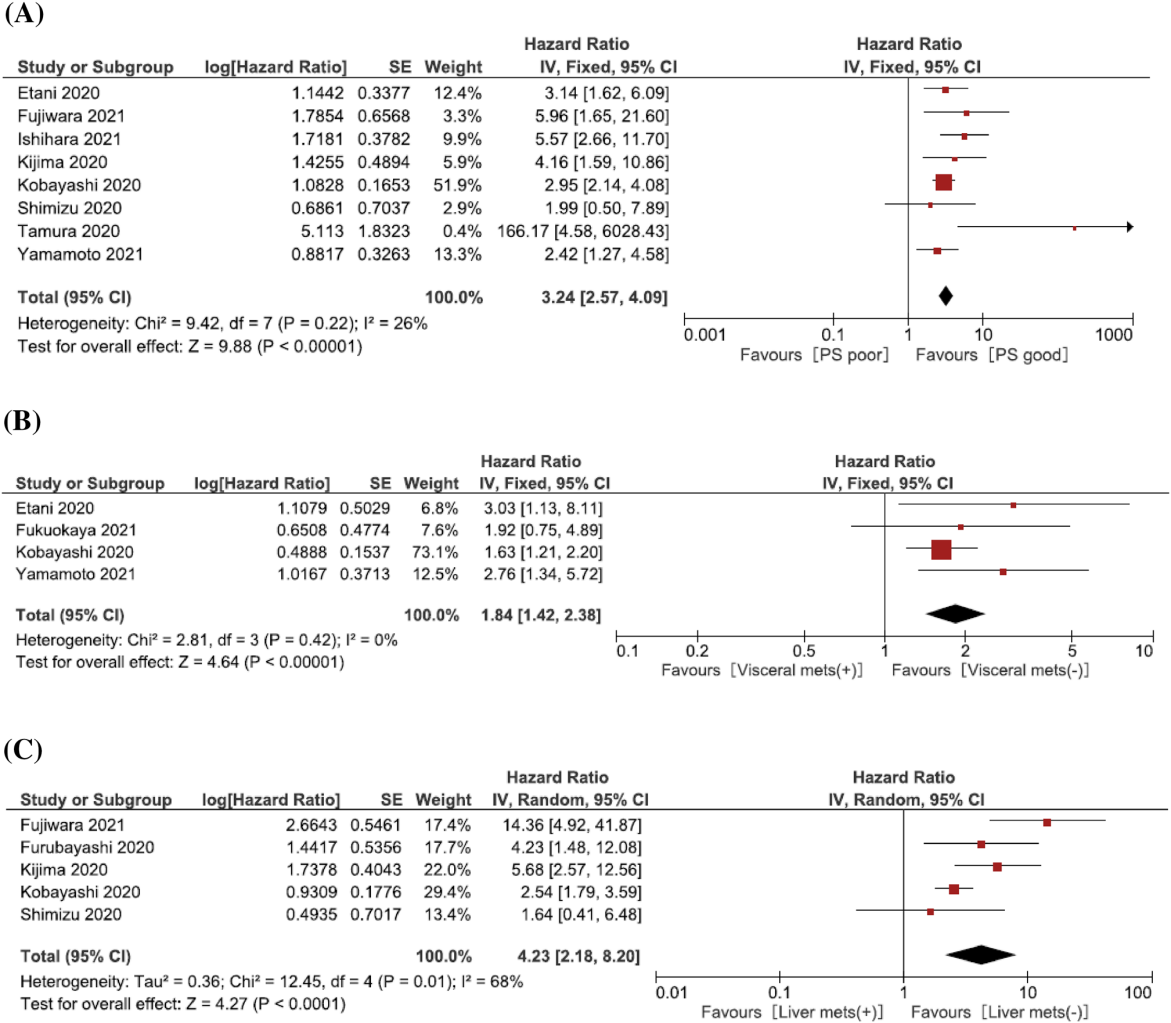

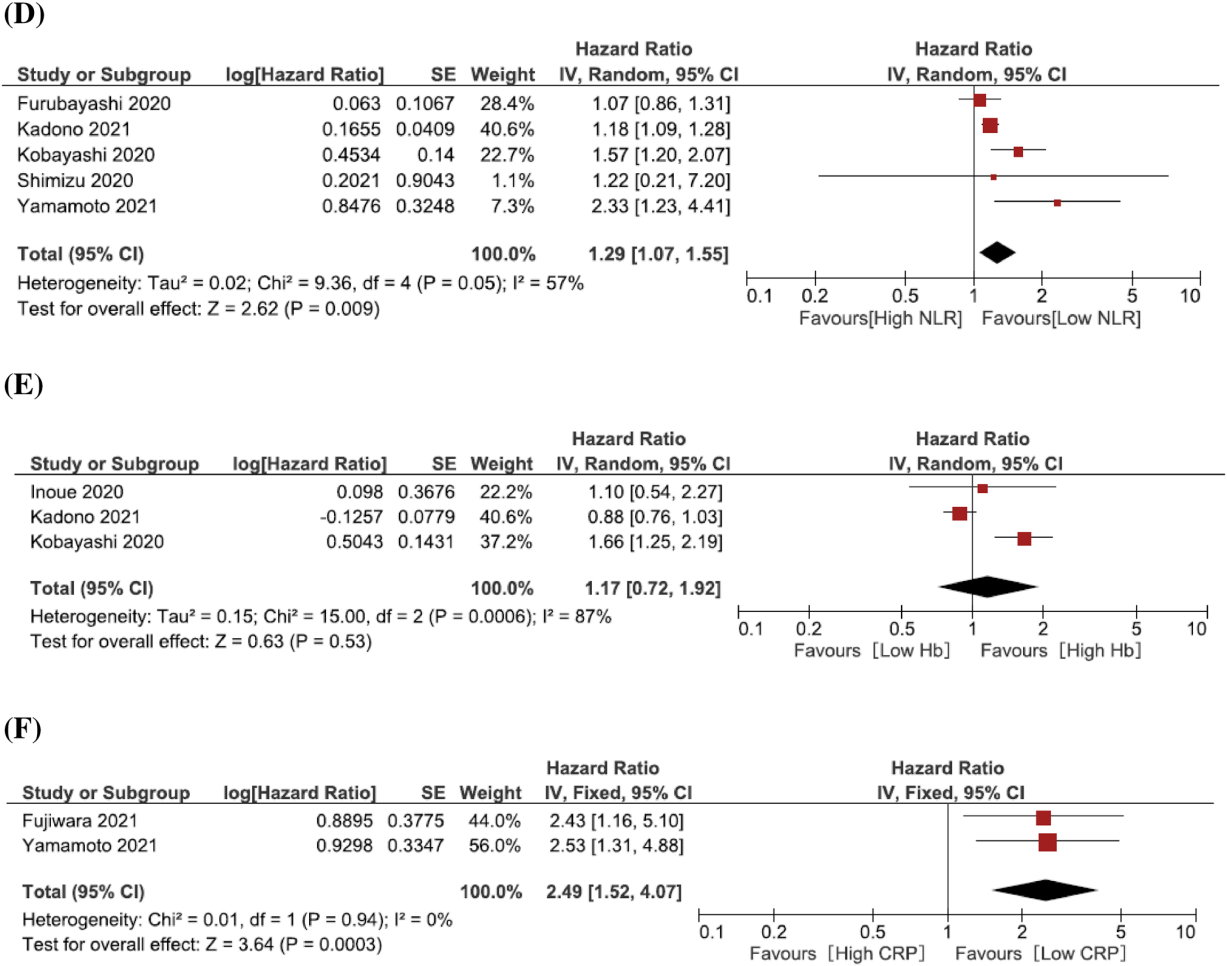
Forest plot (association of clinical features and hematologic biomarkers with overall survival). **A** ECOG-PS; **B** Visceral metastasis; **C** Liver metastasis; **D** Neutrophil–lymphocyte ratio; **E** Hemoglobin; **F** C-reactive protein

Ten studies provided data on the association of Eastern Cooperative Oncology Group Performance Status (ECOG-PS) with OS in 2nd line metastatic UCs treated with pembrolizumab. Eight studies defined the patients’ cut-off as PS ≥ 2. 940 patients were analyzed. The forest plot (Fig. 2a) revealed that ECOG-PS ≥ 2 was significantly associated with worse OS (pooled HR: 3.24, 95% CI 2.57–4.09; *z* = 9.88). The Cochrane’s *Q* test (Chi^2^ = 9.42; *P* = 0.22) and *I*^2^ test (*I*^2^ = 80.5%) revealed no significant heterogeneity. The funnel plot seemed symmetry and did not identify any studies over the pseudo-95% CI (Supplementary Fig. 2A).

#### Association of metastatic site with OS in mUC treated with pembrolizumab

Four studies, including 731 patients, provided data on the association of visceral metastasis with OS in 2nd line metastatic UCs treated with pembrolizumab. The forest plot (Fig. 2b) revealed that visceral metastasis was significantly associated with worse OS (pooled HR: 1.84, 95% CI 1.42–2.38; *z* = 4.64). The Cochrane’s *Q* test (Chi^2^ = 2.81; *P* = 0.42) and *I*^2^ test (*I*^2^ = 0%) revealed no significant heterogeneity. The funnel plot did not identify any studies over the pseudo-95% CI (Supplementary Fig. 2B).

Five studies, including 695 patients, provided data on the association of liver metastasis with OS in 2nd line metastatic UCs treated with pembrolizumab. The forest plot (Fig. 2c) revealed that liver metastasis was significantly associated with worse OS (pooled HR: 4.23, 95% CI 2.18–8.20; *z* = 4.27). The Cochrane’s *Q* test (Chi^2^ = 12.45; *P* = 0.01) and *I*^2^ test (*I*^2^ = 68%) revealed significant heterogeneity. The funnel plot identified one study over the pseudo-95% CI (Supplementary Fig. 2C).

#### Association of NLR with OS in mUC treated with pembrolizumab

Five studies, including 777 patients, provided data on the association of neutrophil–lymphocyte ratio (NLR) with OS in metastatic UCs treated with pembrolizumab. The forest plot (Fig. 2d) revealed that pretreatment high NLR was significantly associated with worse OS (pooled HR: 1.29, 95% CI 1.07–1.55; *z* = 2.62). The Cochrane’s *Q* test (Chi^2^ = 9.36; *P* = 0.05) and *I*^2^ test (*I*^2^ = 57%) revealed significant heterogeneity. The funnel plot identified one study over the pseudo-95% CI (Supplementary Fig. 2D).

#### Association of Hb with OS in mUC treated with pembrolizumab

Three studies, including 627 patients, provided data on hemoglobin (Hb) association with OS in 2nd line metastatic UCs treated with pembrolizumab. The forest plot (Fig. 2e) revealed that a low pretreatment Hb level was not associated with OS (pooled HR: 1.17, 95% CI 0.72–1.92; *z* = 0.63). The Cochrane’s *Q* test (Chi^2^ = 15.00; *P* = 0.0006) and *I*^2^ test (*I*^2^ = 87%) revealed significant heterogeneity. The funnel plot identified one study over the pseudo-95% CI (Supplementary Fig. 2E).

#### Association of CRP with OS in mUC treated with pembrolizumab

Two studies, including 195 patients, provided data on c-reactive protein (CRP) association with OS in 2nd line metastatic UCs treated with pembrolizumab. The forest plot (Fig. 2f) revealed that pretreatment CRP was significantly associated with worse OS (Pooled HR: 2.49, 95% CI 1.52–4.07; *z* = 2.62). The Cochrane’s *Q* test (Chi^2^ = 0.01; *P* = 0.94) and *I*^2^ test (*I*^2^ = 0%) revealed no significant heterogeneity. The funnel plot did not identify any studies over the pseudo-95% CI (Supplementary Fig. 2F).

### Other factors associated with OS

As for hematological biomarkers, high pretreatment level of LDH was significantly associated with worse OS [36]. In addition to high pretreatment level of NLR and CRP, percentage changes in these levels after initiation of pembrolizumab treatment were also significantly associated with OS in one study each [42, 46]. UTUC [40] and smoking history/exposure [37, 43] as pretreatment patients’ characteristics were significantly associated with OS. As for systemic nutritional condition, Geriatric Nutritional Risk Index [35]: a nutritional assessment tool defined by serum albumin levels and the ratio of actual to ideal body weight, Prognostic Nutritional Index[40]: a prognostic model comprising serum lymphocyte counts and albumin, and sarcopenia[45] were all significantly associated with OS.

## Discussion

Despite large progress effected by pembrolizumab in patients with metastatic UCs, the rate and length of ORR and OS benefits are still not satisfactory [3, 5]. Identification of predictive biomarkers and prognostic factors is indispensable for precise and patient-centered clinical decision-making. This systematic review and meta-analysis suggests that poor performance status (PS), visceral (particularly liver) metastasis, high pretreatment level of NLR and CRP are all associated with OS.

ECOG-PS has been used as a tool to guide clinicians regarding fitness for systemic therapy [48]. It has been shown to be an independent prognostic factor for OS in patients with advanced melanoma treated with ICIs [49] and advanced/metastatic UC treated with chemotherapy [26, 31]. In a recent retrospective cohort study focused on association with ECOG-PS and survival in advanced UCs patients treated with ICIs, OS was shorter in patients with ECOG-PS ≥ 2, particularly in the 1st line setting [50]. Our analysis suggests that ECOG-PS ≥ 2 was significantly associated with OS in platinum-refractory metastatic UC patients treated with pembrolizumab. Parikh et al. reported that although mortality seemed to be favorable after approval of ICIs, the use of ICIs at the end of life in patients with metastatic UCs has been rising dramatically [51]. The establishment of guidelines and policy implications for treating poor PS patients with ICIs is mandatory.

Regarding the site of metastasis, visceral metastasis and especially liver metastasis, are associated with worse OS. The presence of liver metastases has been previously reported to be a poor prognostic factor for the patients treated with chemotherapy [3, 31]. In the subgroup analysis of the phase 3 trial, KEYNOTE-045, liver metastasis was associated with worse OS in patients treated with pembrolizumab or chemotherapy [3]. In agreement with this study, our analysis confirmed liver metastasis to confer a significantly higher risk of ICI therapy failure.

Recently, pretreatment hematologic inflammation biomarkers such as NLR have been shown to prognosticate ICI response in various cancers alone or in combination with other predictors for these patients [52–57]. Moreover, hematologic markers, such as NLR, have been suggested as biomarkers for progression after radical cystectomy [58]. However, it remains controversial whether pretreatment NLR provides prognostic information for identifying clinical responses to pembrolizumab in platinum-refractory metastatic UC patients. Previous studies have shown that a high neutrophil count was correlated with a decreased number of CD8-positive T cells [59], and the increased infiltration of lymphocytes in the tumor region was associated with a better response to ICIs [60]. Furthermore, it is credible that alterations of circulating lymphocytes could be associated with the efficacy of ICIs [61], because they could enhance antitumor immunity by blocking negative regulators of T-cell function [62].

The elevation of CRP levels is a representative acute phase reactant that is widely used to evaluate systemic inflammation. The correlation between inflammation and malignant potential is widely known [63]. The elevation of CRP levels has been reported as a poor predictor of advanced UCs [64] and metastatic melanoma treated with ICIs [49, 65, 66]. In the present study, CRP and NLR, which might be affected in the tumor microenvironment by immune cells, were associated with worse OS. Pretreatment CRP may also help, along with other markers in a model, to guide clinical decision-making for ICIs, as it is likely to reflect the biology of the tumor and/or its microenvironment[67].

In the chemotherapy era, Sonpavde et al. demonstrated that serum albumin was externally validated as a prognostic factor for OS in addition to Hb, ECOG-PS, and liver metastasis in advanced UC patients with ten prospective phase II trials of salvage systemic agent therapy following platinum-based chemotherapy [31, 68]. Our findings are consistent with previous studies and could confirm the utility of these prognostic factors in the ICI treatment era; furthermore, we added the importance of inflammatory markers such as NLR and CRP as promising biomarkers for mUC patients treated with ICIs.

Finally, the most recent study using patient-level data from phase I/II trials to build a prognostic model for metastatic UCs treated with atezolizumab, demonstrated that ECOG-PS, liver metastasis, platelet count, NLR, and LDH are factors for prognosticating OS [69]. Together with our findings, we conclude that ECOG-PS, liver metastasis, and NLR are essential prognostic factors in patients of metastatic UCs treated with PD-1/PD-L1 inhibitors.

Although we found a strong association between several clinical/hematologic characteristics and mortality in 2nd line metastatic UC patients treated with pembrolizumab, our study suffers from several limitations that need to be taken into account. First, statistical analysis for assessing funnel plots was not performed due to a small number of included studies, but reporting bias could have led to the non-publication of negative results. All the studies included were retrospective in design, thus increasing the risk of selection bias. Second, unknown pretreatment factors (e.g., nutritional deficiencies, comorbidities, medications, and lifestyle factors) may have affected the hematologic biomarkers, thus producing systematic bias. Third, there was no established definition of cut-off values for hematologic biomarkers among the studies evaluated. Most investigators chose the cut-off value based on statistical methods, the lower or higher limit of standard or pre-defined biomarker cut-off values in the literature. Fourth, all the studies included were from Asia, Japan; thus, the interpretation of this study might not be reflective for patient of the whole world. Finally, heterogeneity was detected in the OS analysis; thus, the value of these results is limited. Although the random effect model was used to address heterogeneity among the studies evaluated, the conclusions should be carefully interpreted.

## Conclusions

In 2nd line metastatic UC patients treated with pembrolizumab after platinum-based systemic chemotherapy, patient characteristics with poor PS and visceral metastasis, particularly liver metastasis, were associated with worse OS. Furthermore, pretreatment high NLR and CRP were blood-based prognosticators of OS. Our findings might help to guide the prognostic tools for clinical decision-making; however, they should be interpreted carefully, owing to limitations regarding the retrospective nature of primary data. Further investigation is mandatory to explore these and other biomarkers to build a reliable, generalizable, accurate, and easy-to-use predictive tool.

## Supplementary Information

Below is the link to the electronic supplementary material.

Supplementary file1 (DOCX 37 KB)

Supplementary Fig. 1 PRISMA 2009 Checklist (DOCX 29 KB)

Supplementary Fig. 2 Funnel plot (association of clinical features and hematologic biomarkers with overall survival). (A) ECOG-PS; (B) Visceral metastasis; (C) Liver metastasis; (D) Neutrophil–lymphocyte ratio; (E) Hemoglobin; (F) C-reactive protein (DOCX 111 KB)

## Data Availability

All data generated or analyzed during this study are included in this published article.

## Abbreviations

CI: Confidence Intervals
ECOG-PS: Eastern Cooperative Oncology Group Performance Status
Hb: Hemoglobin
HR: Hazard Ratio
ICI: Immune Checkpoint Inhibitors
IQR: Interquartile Range
LDH: Lactate Dehydrogenase
NA: Not Applicable
NLR: Neutrophil Lymphocyte Ratio
OS: Overall Survival
ORR: Objective Response Rate
PD-1: Programmed cell Death protein 1
PD-L1: Programmed Death-Ligand 1
PS: Performance Status
RCTs: Randomized Control Trials
UC: Urothelial Carcinoma
UTUC: Upper urinary Tract Urothelial Carcinoma
