# Supplementary material for: Pretreatment clinical and hematologic prognostic factors of metastatic urothelial carcinoma treated with pembrolizumab: a systematic review and meta-analysis
Source: Int J Clin Oncol. 2021 Nov 10;27(1):59–71. doi: 10.1007/s10147-021-02061-0 (PMC8732925; doi:10.1007/s10147-021-02061-0)
Supplement: Supplementary file 1 — Supplementary file1 (DOCX 37 KB) [file 10147_2021_2061_MOESM1_ESM.docx]

| Risk of bias assessment for NRCTs (ROBINS-I) | | | | | | | | | | |
| --- | --- | --- | --- | --- | --- | --- | --- | --- | --- | --- |
| Study | **Year** | **Confounding** | **Participants’ selection** | **Classification of interventions** | **Deviations from  intended intervention** | **Missing data** | **Measurement of outcomes** | **Selection of the reported result** | **Overall** |  |
| Etani | 2020 | Low | Moderate | Low | Moderate | Low | Moderate | Low | Moderate |  |
| Fujiwara | 2021 | Low | Moderate | Low | Moderate | Low | Moderate | Low | Moderate |  |
| Furubayashi | 2020 | Low | Moderate | Low | Moderate | Low | Moderate | Low | Moderate |  |
| Kobayashi | 2020 | Low | Moderate | Low | Low | Low | Low | Low | Low |  |
| Ogihara | 2020 | Low | Moderate | Low | Moderate | Low | Moderate | Low | Moderate |  |
| Shimizu | 2020 | Low | Moderate | Low | Moderate | Low | Moderate | Low | Moderate |  |
| Tamura | 2020 | Moderate | Moderate | Low | Moderate | Low | Moderate | Low | Moderate |  |
| Yamamoto | 2021 | Moderate | Moderate | Low | Moderate | Low | Moderate | Low | Moderate |  |
| Kijima | 2020 | Moderate | Moderate | Low | Moderate | Low | Moderate | Low | Moderate |  |
| Kadono | 2021 | Low | Moderate | Low | Moderate | Low | Serious | Low | Serious |  |
| Fukuokaya | 2021 | Low | Moderate | Low | Moderate | Low | Moderate | Low | Moderate |  |
| Inoue | 2020 | Low | Moderate | Low | Moderate | Low | Moderate | Low | Moderate |  |
| Ishiyama | 2021 | Moderate | Moderate | Low | Moderate | Low | Moderate | Low | Moderate |  |
| NRCTs: non-randomized comparative studies, ROBINS-I: Risk Of Bias In Non-Randomized Studies -of Interventions | | | | | | | | | | |
