# Supplementary figures and images for: Pretreatment clinical and hematologic prognostic factors of metastatic urothelial carcinoma treated with pembrolizumab: a systematic review and meta-analysis
Source: Int J Clin Oncol. 2021 Nov 10;27(1):59–71. doi: 10.1007/s10147-021-02061-0 (PMC8732925; doi:10.1007/s10147-021-02061-0)

Supplementary Figure 2

(A)


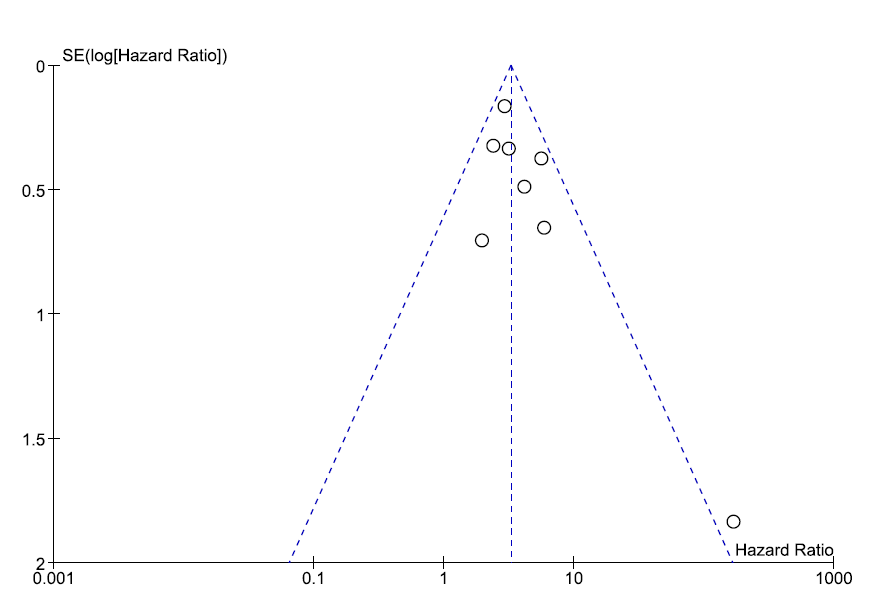


(B)


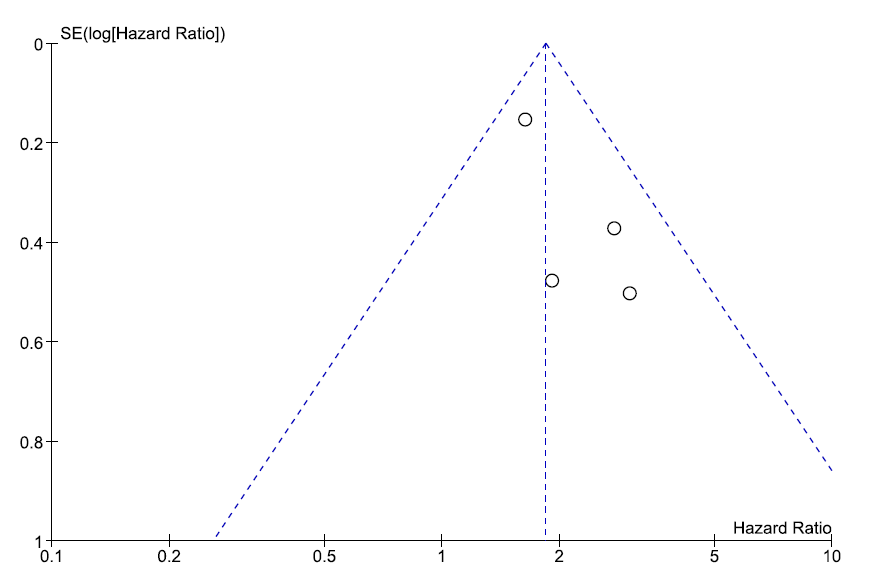


(C)


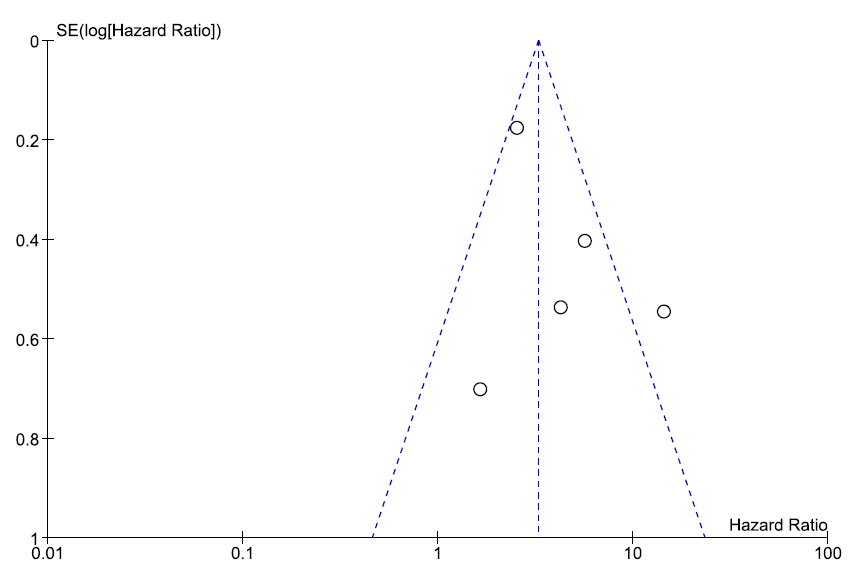


(D)


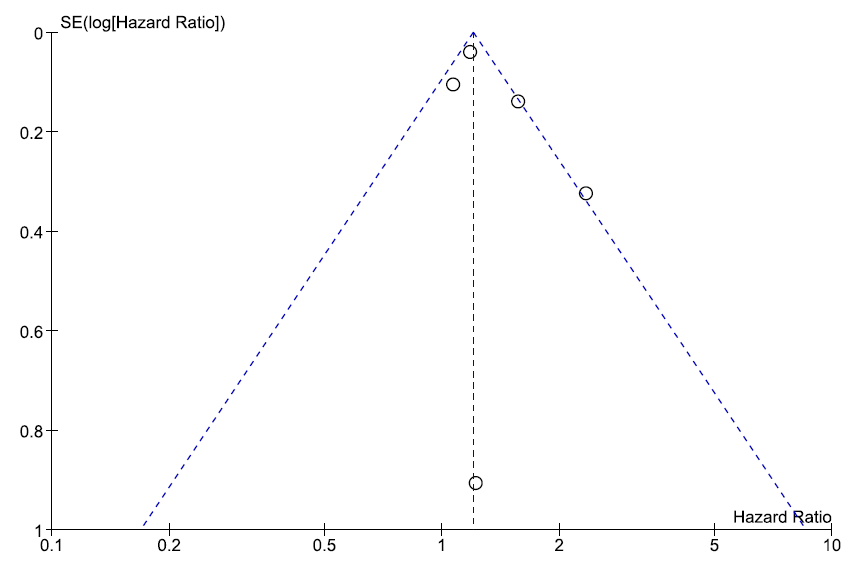


(E)


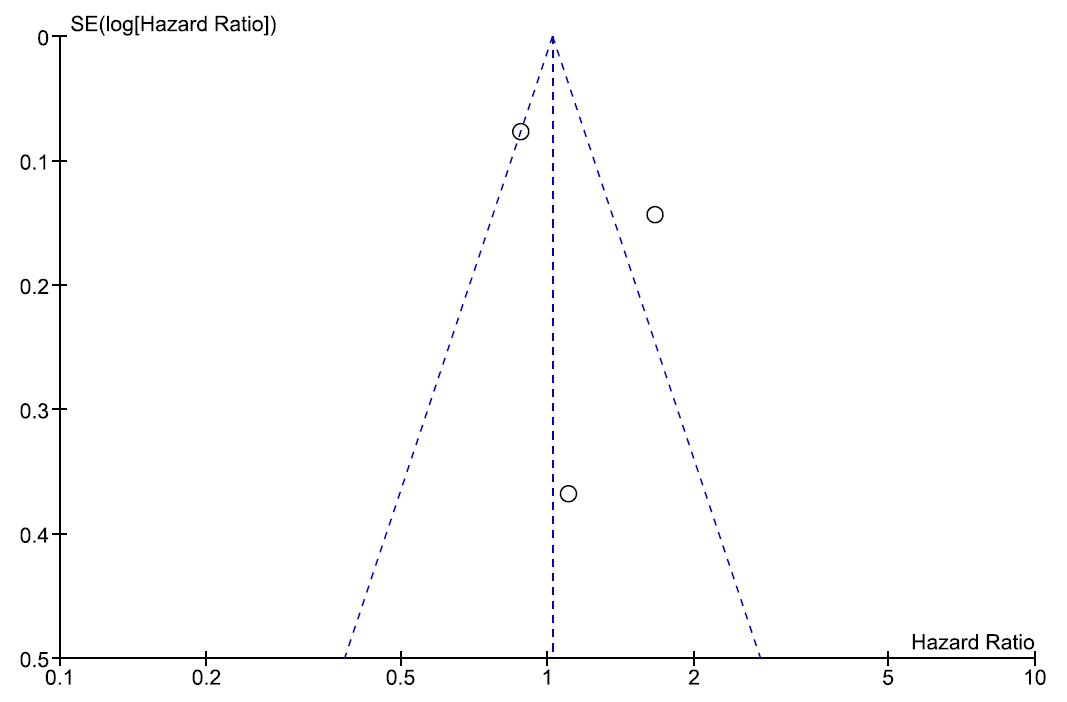


(F)


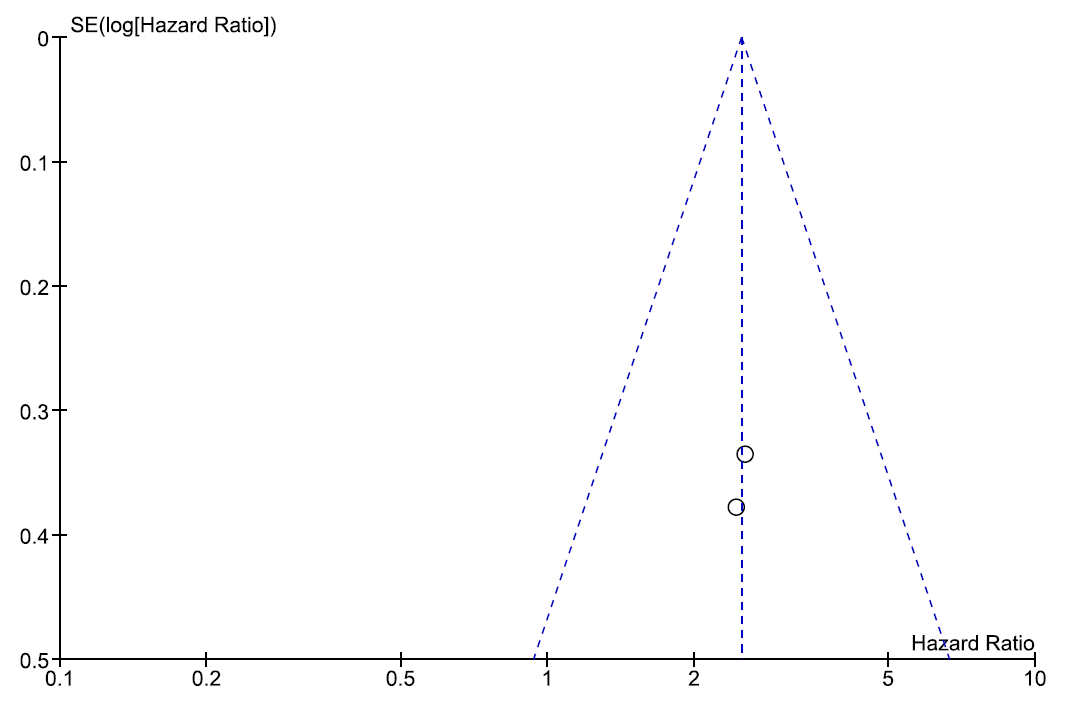

Supplement: Supplementary file 3 — Supplementary Fig. 2 Funnel plot (association of clinical features and hematologic biomarkers with overall survival). (A) ECOG-PS; (B) Visceral metastasis; (C) Liver metastasis; (D) Neutrophil–lymphocyte ratio; (E) Hemoglobin; (F) C-reactive protein (DOCX 111 KB) [file 10147_2021_2061_MOESM3_ESM.docx]
